# Self-medication with antibiotics among non-medical university students of Karachi: a cross-sectional study

**DOI:** 10.1186/2050-6511-15-74

**Published:** 2014-12-23

**Authors:** Syed Jawad Shah, Hamna Ahmad, Rija Binte Rehan, Sidra Najeeb, Mirrah Mumtaz, Muhammad Hashim Jilani, Muhammad Sharoz Rabbani, Muhammad Zakariya Alam, Saba Farooq, M Masood Kadir

**Affiliations:** Aga Khan University, Karachi, Pakistan; Department of Community Health Sciences, Aga Khan University, Karachi, Pakistan

**Keywords:** Self-medication, Antibiotics, Antibiotics resistance, Non-medical, Adverse effects

## Abstract

**Background:**

The prevalence of self -medication with antibiotics is quite high in developing countries as opposed to developed countries. Antibiotics are often taken erroneously for certain ailments, without having the appropriate knowledge of their use. This carries potential risks for the individual as well as the community, in form of several side effects such as antibiotic resistance. Therefore the prevalence of self-medicated antibiotics in developing countries needs to be studied.

**Methods:**

A descriptive cross-sectional study was carried out at six different non-medical universities of Karachi. 431 students were included in the study. Data was collected using self-administered questionnaires and analyzed using SPSS version 19.

**Results:**

50.1% students reported having self-medicated themselves in the past 6 months and 205 (47.6%) reported self-medication with antibiotics. Amoxicillin was the most self-prescribed antibiotic (41.4%). Awareness of the adverse effects of antibiotics was demonstrated by 77.3% of the students and sleep disturbance was the most commonly known (46.5%) side effect. 63.1% denied having any knowledge about antibiotic resistance and only 19.9% correctly knew that indiscriminate use of antibiotics can lead to increased antibiotic resistance.

**Conclusion:**

The prevalence of self-medication with antibiotics among the non-medical university students was high despite the awareness of adverse effects. Antibiotic resistance was a relatively unknown terminology.

**Electronic supplementary material:**

The online version of this article (doi:10.1186/2050-6511-15-74) contains supplementary material, which is available to authorized users.

## Background

Self-medication can be defined as the use of non-prescription medicines by people on their own initiative. The definition can be expanded to include treatment of family members and dependents, in particular children/minors and the elderly [1]. Self-medication is a component of self-care and is considered as primary public health resource in health care system.

Studies have revealed the burden of self-medication with antibiotics to be higher in developing countries than in developed countries [2]. The prevalence has been reported to be 3% in northern Europe as compared to the 4-75% in Asia [3]. One study done in Karachi, Pakistan, showed the frequency of self-medication (as a whole) among university students to be as high as 80.4%. Frequency among non-medical students was 83.3% while for those in medical school, it was 77.7% [4].

World Health Organization has mentioned, according to a survey that self-medication, if administered appropriately and responsibly can help prevent and treat diseases economically and without medical consultation [5].

Although self-medication may prove useful when used judiciously, it is more often used erroneously, without proper guidance and rationale. This fact is highlighted by a study conducted in Jordan, which showed that 67.1% of adults believed that antibiotics cure common cold and cough [6]. Medications administered inappropriately not only leads to wastage of resources but also carries potential serious and life-threatening adverse effects for the users [3, 6]. Research of health seeking attitudes in different parts of the world reveals that self-medication (with any drug) is higher among the literate, the young and people in low and middle income countries. The same study also highlights that 82.5% of the students are aware of some form of harm caused by self-medication [7–9].

The misuse of antibiotics is a serious problem in several respects. The medical community may be aware of the seriousness of this rising problem but this is not reflected in the non-medical community, which is still relatively unaware of any such issue. This was exhibited in one study in Italy, which showed that only 9.8% of the general public knew the definition of antibiotic resistance and only 21.2% knew when was it appropriate to use antibiotics [2]. Another study conducted in Yemen and Uzbekistan showed that 49% of respondents discontinued antibiotics as soon as they started feeling better [10].

The developing world is the hub for the emergence of rapidly mutating and resistant strains of several pathogens, including S. pnemoniae, S. typhi, and Shigella species [11–13]. Emergence of antibiotic resistant strains of several pathogens is linked directly to the use of antibiotics and with their unregulated use (or misuse) [11]. Secondly, unregulated use of antibiotics results in improper dosing (over and under dosing), which may adversely affect the person being administered the drug. Thirdly, antibiotics have their own side effects and hazards, which need to be considered, in particular, for people with other co-morbids.

Public education and enforcing and implementing laws about prescribed medications can help decrease the rate of self-medication as shown by previous researches in the developed world [3, 14]. Therefore, ideally, antibiotics need to be regulated via prescription-only sales. Legally, the sale of antibiotics is regulated on a ‘sold on prescription only’ basis and this needs to be put into effect in reality as well [5, 15]. So for implementation of such policies and carrying out general awareness programs, information should be available about how common the use of self-medicated antibiotics is. Therefore, we aimed to provide the prevalence of self-medication with antibiotics amongst the university students of Karachi, who are not associated with health care or medicine.

## Methods

### Study design and setting

This study was a descriptive cross-sectional study where the prevalence of self-medicated antibiotics among the university students of Karachi was investigated. The study was conducted in six different non-medical universities of Karachi, which included two engineering universities, one business and management university, one art university and two multi-disciplinary universities.

### Sample size calculation

Previous studies in Karachi showed the prevalence of self-medication to be 75% - 80% [4]. Hence using 80% as a reference prevalence of use of self-medicated antibiotics in university students of Karachi, the sample size was calculated to be 246, at confidence level of 95%. For the second objective of this study, prevalence of each risk factor from previously conducted researches was used, which generated a sample size of 400. Lastly, prevalence of knowledge of adverse effects was taken to be 62%, and the aggregate sample size was calculated to be 423 after 10% inflation. Therefore, 425 individuals were planned to be approached and by the end, a total of 431 students answered the questionnaire (Additional file 1).

### Inclusion and exclusion criteria

All male and female students enrolled in undergraduate or postgraduate programs in universities of Karachi who understood English were included. Medical, paramedical and pharmacology students were excluded from the study.

Self-medication was defined as use of any medication, in the past 6 months, with one’s own accord, which was not prescribed by a doctor. A time period of 6 months was chosen to eliminate recall bias amongst those who had used antibiotics and were likely to recall it in this adequate time period, with those who had not used antibiotics and would also clearly remember not having used them.

### Data collection procedure

A convenience sampling method was used to complete the required sample size. Students were approached in the areas specified by the respective university managements. Informed consent, both verbal and written, was taken from the study volunteers. The self-administered questionnaire was then filled by the students and returned back to our questionnaire administrators.

### Date collection tool

A paper based questionnaire was used for data collection. The questionnaire was divided into three sections (A, B and C), each of which was preceded by statements to clarify the nature of questions that would follow, for the benefit of the subject. Details of this questionnaire are as follows: Section A, containing 6 items, assessed subject demographics, including age, sex, year in university, health insurance, household income. These were all close ended questions. Data is reflected in results Table 1, which is a summary of the data collected in this section. Section B, containing 7 items, assessed frequency of use of self-medicated antibiotics in the past 6 months along with the type of antibiotic used. A brief description of the definition of self-medication preceded the start of this section. Subjects were thence asked about self-medication in the past 6 months in general and then with respect to antibiotics alone. Subjects were given a list of generic along with market names of some of the most commonly used antibiotics. However, an additional option of ‘other antibiotics used’ was also presented for the ease of the subject and was the only open ended option in this section. Data is reflected in Table 2, which is a summary of the data collected in this section. Lastly, Section C assessed knowledge of adverse effects of antibiotics. This section contained questions, where subjects were asked to check as many adverse effects they thought were possible, with the option ‘others’ as the only open ended question. Knowledge of the term antibiotic resistance and knowledge about effect of antibiotic use on resistance was assessed using close ended questions. The data from this section is reflected in Table 3.

**Table 1:** **Distribution of demographic factors among non-medical students of Karachi**

	Students using self-medication (n = 205)		Students not using self-medication (n = 226)		Odds ratio (95% confidence interval)
	Frequency (n)	Percentage (%)	Frequency (n)	Percentage (%)	
Gender					0.903(0.615-1.326)
• Male	123	60	130	57.5	
• Female	82	40	96	42.5	
TOTAL	205		226		
Year in university					
• 1^st^	116	56.6	132	58.4	
• 2^nd^	41	20	39	17.3	
• 3^rd^	16	7.8	21	9.3	
• 4^th^	29	14.1	29	12.8	
• 5^th^	1	0.5	3	1.3	
• >5^th^	2	1	2	0.9	
Marital status					
• Single	197	96.1	214	94.7	
• Married	3	1.5	8	3.5	
• Divorced	5	2.4	4	1.8	
Household income					
• <50,000	51	26.8	64	30.9	
• 50,000 to <100,000	73	38.4	66	31.9	
• 100,000-150,000	38	20	32	15.5	
• >150,000	28	14.7	45	21.7	
Healthcare expenses covered					1.186(0.804-1.750)
• Yes	116	57.4	136	61.5	
• No	86	42.6	85	38.5

**Table 2:** **Shows frequency of self-medicated antibiotics among non-medical university students of Karachi**

	Used once (n = 127)	Used twice (n = 51)	More than twice (n = 54)
Ciprofloxacin	16(7.6%)	6(2.9%)	5(2.4%)
Cotrimoxazole	12(5.7%)	3(1.4%)	5(2.4%)
Amoxicillin	47(22.4%)	19(9%)	21(10%)
Ampiclox	7(3.3%)	3(1.4%)	3(1.4%)
Ampicillin	4(1.9%)	1(0.5%)	2(1.0%)
Erythromycin	8(3.8%)	3(1.4%)	3(1.4%)
Metronidazole	33(15.7%)	16(7.6%)	15(7.1%)

**Table 3:** **Knowledge of adverse effects caused by antibiotics**

Symptoms	Frequency (n)	Percentage (%)
Diarrhea/abdominal pain	99	23.1
Nausea/Vomiting	127	29.7
Allergic Reactions	166	38.8
Yellow eyes/skin	51	11.9
Tiredness/Dizziness	128	29.9
Headache	143	33.4
Fever	79	18.5
Kidney problems	64	15
Liver problems	47	11
Teeth discoloration	36	8.4
Muscle/joint pain	65	15.2
Numbness/tingling	35	8.2
Sleep problems	199	46.5

The tool underwent a pilot phase where 30 subjects who met the inclusion criteria were asked to respond to the questionnaire. Data collected was analyzed and feedback was taken from the subjects about possible difficulties faced. No difficulties were faced by either subject or researcher. However, the data from these 30 subjects was not included in the study sample or result.

### Data entry and analysis

Epidata 3.1 was used for data entry. Data analysis was performed using SPSS version 19. The software was used to run a descriptive analysis and the frequency tables generated were used to calculate the prevalence of self-medication. Frequency of use of different classes of antibiotics and frequency of the reasons which led to use of self-medicated antibiotics was also calculated using descriptive analysis. Chi- square test of association was conducted for association of factors between self-medicated antibiotic users and non-users. We also calculated prevalence of awareness of side effects and frequency of most common adverse effects experienced by participants. Finally, frequency of knowledge regarding antibiotic resistance was also calculated.

### Ethical consideration

Ethical clearance was given by Ethical Review Committee of Aga Khan University. Prior permission was sought from all 6 universities before conducting surveys among their students. Informed consent forms were signed by the study participants. Confidentiality was maintained by separating the filled questionnaires with the consent form.

## Results

### Study population

Four hundred and thirty one (431) non-medical University Students from Karachi, Pakistan were surveyed. Mean age of these students was 20.04(±1.74) years. The proportion of males was 58.7% while that of females was (41.3%). and majority (57.5%) was first year university students. Of the 431 University students surveyed, merely 397 gave information about their household income. About 64% of these respondents had a household income of <100,000 PKR.

### Prevalence of self-medication and its association with demographic factors

205 respondents (47.6%) reported using antibiotics not prescribed by a doctor in the last six months. There was no statistical significance for frequency of self-medication with antibiotics in relation to sex, marital status, year of university, household income and having healthcare expenses covered, as demonstrated by Table 1.

### Trends in antibiotic usage

The most common antibiotic used for self-medication purposes was amoxicillin which was used by 81(41.4%) study participants. Amoxicillin was followed by Metronidazole (30.5%), Ciprofloxacin (12.7%) and Cotrimoxazole (9.5%). Erythromycin and Ampicillin/cloxacillin were used by 6.7% and 6.2% of the sample population respectively and other antibiotics were amongst the less frequently used antibiotics. The frequency of use of each antibiotic is depicted in Table 2.

Forty-three (10%) students mistook other classes of drugs for antibiotics. This was assessed using the ‘Other antibiotics’ option in Section B, where subjects were asked to enumerate any antibiotic other than the ones in the list provided by the researchers that they might have used. The most commonly mistaken drugs were analgesics and anti-allergy medications.

42.8% of students admitted to choosing the antibiotics themselves, 76% percent believed that they knew what antibiotics were and 77.3% of the population knew that antibiotics cause adverse effects.

Of the 217 students who reported using antibiotics, 83 used them to relieve fever (41.1%), 81 for pain relief (40.1%) while 80 students reported use of antibiotic for relief from respiratory symptoms (39.6%). Use for relief from gastrointestinal problems and from urinary symptoms was 15.8% and 6.9% respectively.

**Figure 1:**
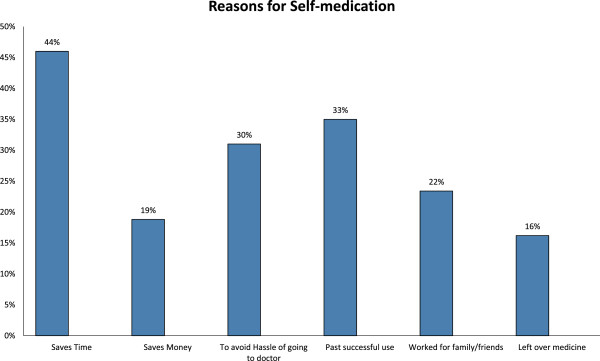
**Factors leading to the use of self-medicated antibiotics in non-medical university students of Karachi.** Percentages of non-medical students stating each factor as the cause for the use of self-medicated antibiotics.

The reasons for choosing to self-medicate with antibiotics rather than visiting a certified healthcare professional for their ailments by students (N = 211) have been reported in Figure 1.

Out of the 427 people surveyed who answered the question about adverse effect awareness, 330 were aware that antibiotic use could lead to adverse effects (77.3%).

The most common adverse effects that the students knew could be caused by antibiotics were sleep problems (46.5%) and allergic reaction (38.8%). Other adverse effects that students showed most awareness about were headaches (33.4%), tiredness or dizziness (29.9%), nausea/ vomiting (29.7%), and diarrhea/ abdominal pain (23.1%). The complete list of adverse effects thought to be caused by antibiotics by University students is presented in Table 3.

However, only 21.8% of the students (93 out of 427) reported experiencing adverse effects after antibiotic usage. Of the 71 students who specified which adverse effects they had experienced, 19 reported presence of abdominal complaints after antibiotic usage (26.7%), 13 reported an allergic reaction (18.3%), 9 reported sleep disturbance (12.7%), and 8 reported weakness (11.3%).

When questioned regarding the term ‘antibiotic resistance’, 20.9% of the 425 students reported having heard of it while 63.1% denied having any knowledge about the term. However, as shown in Table 4, only 83 out of 423 students (19.9%) correctly knew that indiscriminate use of antibiotics can lead to increased antibiotic resistance. Majority (64.3%) admitted to having no idea about the relation of antibiotic resistance to indiscriminate antibiotic use.

**Table 4:** **Knowledge of effect of inadequate use of antibiotics on antibiotic resistance**

Effect on antibiotic resistance	Frequency (n)	Percentage (%)
Increases	84	19.9
Decreases	45	10.6
Remains the same	22	5.2
I don’t know	272	64.3

An interesting fact to note is that 60.3% of the students (n = 123) who had self-medicated with antibiotics reported having heard of antibiotic resistance, showing a significant association between the two (*χ*^2^ = 1.409, p = 0.494).

## Discussion

The prevalence of self-medication with antibiotics, as previously mentioned, is much higher in developing countries as compared to developed ones [2]. This study focused in particular on the non-medical university students.

Our study found out that almost half of our study population had self-medicated with antibiotics in the last 6 months. No study was available for comparison in Pakistan which had studied the prevalence of self-medication with antibiotics. However, in two studies, the prevalence of self-medication as a whole amongst university students of Karachi was found to be around 80% [4, 9]. The possible reasons for this difference could be that our study included self-medication with antibiotics specifically and that too, within the last six months only, whereas the aforementioned studies did not restrict the time period for recall.

In other countries, studies have shown prevalence of self-medication with antibiotics to be 47.8% in Southern China, 79.5% in Sudan and 48% in Iran [16–18]. Similar trends for prevalence have also been found in studies conducted for general community in Middle Eastern countries [10].

Our study also assessed various factors that could be associated with the use of self-prescribed antibiotics amongst the study population. No association could be established between the demographic factors such as gender, year of study, marital status, monthly family income, health coverage and self-medication with antibiotics. These findings are consistent with the previous studies which found no association between self-medication in general and socio-demographic factors [19].

Antibiotics carry several risks when used without physician advice. The exact burden of this problem is not known. The results indicate that the three most common reasons for self-prescription were to save time, previous successful experience and avoiding the hassle at clinics. These findings are not surprising considering the fact that most of the health care expenditure in our country is out-of-pocket and the healthcare facilities are already over-burdened; unable to satisfy the health related needs of the general population. For better understanding of this situation, further studies should be carried out to assess the satisfaction of patients with the health care services, especially the outpatient clinics.

Amongst the list of antibiotics given to the participants the most commonly used antibiotics were amoxicillin and metronidazole. This reflects in another study done in Karachi where the most common antibiotic used for self-medication was metronidazole, followed by co-amoxiclav and amoxicillin respectively [4]. In our study, fever, pain and respiratory complaints were the most common reasons for which the antibiotics were used. This finding matches the common reasons highlighted for self-medication with antibiotics in previous studies [10, 17].

A large proportion (42.8%) of students admitted to choosing the antibiotics themselves. This high percentage might be due to our study subjects being educated university students who had access to online learning resources and may think of themselves as well equipped with knowledge of antibiotics. This is also reflected in our study results which showed that around 76% percent believed that they knew what antibiotics were. Further assessing knowledge about antibiotics, it was found that a vast majority (77.3%) of the population knew that antibiotics cause adverse effects and amongst the participants who had self-medicated with antibiotics, a similar proportion was aware of potential adverse effects.

A very small proportion of the total study population, however, had heard of the term antibiotic resistance and an even less had an idea that resistance increased with indiscriminate use of antibiotics. The rest denied having any knowledge of antibiotic resistance and its varying trend with indiscriminate use. Considering that our study subjects included students from non-medical background only, these findings were expected as antibiotic resistance is a technical term used in the field of medicine. These finding could be used as a basis to discourage self-medication with antibiotics.

Ideally, it is the government’s responsibility to establish that any population uses self-medication responsibly. Drugs that are available without the need for prescription by physician or trained medical personnel should only be the ones which are safe to use. The government should also ensure that users are educated properly about not only the use of the drug but also the correct dosages, duration of use and potential side effects associated with them as antibiotics are tailored not only according to the disease but also according to the individual patient profile.

There are few limitations in our study. We did convenience sampling instead of systematic randomized approach. However, we tried to minimize the effect of this by taking a large sample size and selecting 6 different universities to ensure diversity among our study participants. The questions assessing the knowledge about antibiotics were very subjective so the true picture regarding this could not be determined.

## Conclusion

This study was one of the few studies done in this part of the world to explore the prevalence and practices of self-medication with antibiotics and knowledge about the possible side effects of such practices among non-medical university students of Karachi. The results obtained can help in providing a framework for designing programs that will create awareness about the risks of self-prescribed antibiotics.

The prevalence of self-medication with antibiotics among the non-medical university student was high, with no variation based on gender, year of study, marital status, monthly family income and health. The majority of the study population was aware of potential adverse effects of antibiotics and yet the practice of using self-prescribed antibiotics was seen.

The study suggested that there is a necessity for educational programs emphasizing on the risks associated with indiscriminate antibiotic use in which health care providers, pharmacists and others, including parents, should be actively involved in health education that will help in inculcating the practice of responsible use of antibiotic amongst the general population at an early age. The study also showed that there is a need for strict law enforcement to limit the purchase of antibiotics without a prescription.

## Electronic supplementary material

Additional file 1: **Frequency of self-medicated antibiotics, factors associated with their administration and knowledge of their adverse effects among university students of Karachi.** (DOCX 121 KB)
