# Supplementary material for: Self-medication with antibiotics among non-medical university students of Karachi: a cross-sectional study
Source: BMC Pharmacol Toxicol. 2014 Dec 23;15:74. doi: 10.1186/2050-6511-15-74 (PMC4320599; doi:10.1186/2050-6511-15-74)
Supplement: Supplementary file 1 — Additional file 1: Frequency of self-medicated antibiotics, factors associated with their administration and knowledge of their adverse effects among university students of Karachi. (DOCX 121 KB) [file 40360_2014_362_MOESM1_ESM.docx]

ID Number:

**Frequency of self-medicated antibiotics, factors associated with their administration and knowledge of their adverse effects among university students of Karachi.**

**DEMOGRAPHICS**

1. **What is your age?**

__________ (years)

2. **What is your gender?**

- Male
- Female

3**. In what year of University are you?**

- 1^st^ year
- 2^nd^ year
- 3^rd^ year
- 4^th^ year
- 5^th^ year
- >5^th^ year

4**. What is your marital status?**

- Single
- Married
- Divorced

5. **What is your monthly household income in Pakistani Rupees?**

- <50,000
- 50,000-<100,000
- 100,000-150,000
- >150,000

6**. Are your healthcare expenses covered?**

- Yes
- No

**If Yes,**

- By health Insurance
- By Employer
- Others (please specify

**The next few questions are about Self-medication:**

7**. Have you used any medicine(s) in the last 6 months on your own, which was not prescribed by a doctor (i.e. self-medication)?** (*This also applies to any current medication you are taking*)

- Yes
- No

***If NO, Please Skip to Question No. 14***

***If YES:***

**Does it include any medicine(s) from the below mentioned list AND/OR any other Antibiotics? Please also mention the frequency in the provided table.**
*You may choose more than one option if applicable and please write the name of other antibiotic used in the space provided below*

***Frequency In last 6 months
(please choose one)***

| once | Twice | More than twice |
| --- | --- | --- |

**List of Medicines:**

|  |  |  |
| --- | --- | --- |
|  |  |  |
|  |  |  |
|  |  |  |
|  |  |  |
|  |  |  |
|  |  |  |
|  |  |  |

- Ciprofloxacin (Ciprox, Mercep, Ciproxin, Quinoflox)
- Co-trimoxizole (Septran, Bactrim, Sulfatrim)
- Amoxicillin (Amoxil, Augmentin, Ospamox, Zeemox)
- Ampicillin/Cloxacillin (Ampiclox)
- Ampicillin (Ampicap, Adacillin, Ampicil, Ampcigen)
- Erythromycin (Erythroxin, Emycin, Deltacin, Erymox, D-Mycin)
- Metronidazole (Flagyl, Tamizole, Zavad, Ambizole)
- Other Antibiotics(please specify)

***If you have not used any medicine(s) from the above mentioned list AND/OR any other antibiotic, please skip to Question No. 14***

*For Questions 11-14, please include information for all medicines you have mentioned in the previous question, separated by comma*

8. **Do you remember for how long was the above mentioned medicine(s) used?**

- Yes
- No

**If YES, for how may days did you use the medicine(s)?** (Days)

9**. Do you remember the approximate cost for the Medicine(s) you used?**

- Yes
- No

**If YES, what was the cost?** (PKR)

10. **Do you remember the dosage of the above mentioned medicine(s) you used?**

- Yes
- No

**If YES, please specify (e.g. 500mg thrice daily**)

11. **For what symptom was the medicine(s) used? (Please check all that apply)**

- Respiratory symptoms (Cough, cold, sore throat)
- Gastrointestinal symptoms (diarrhea, vomiting, constipation, abdominal pain)
- Urinary complaints (burning/pain during urination, abnormal discharge)
- Fever.
- Pain.
- Others (Please specify)

12**. Why did you choose self-medication with the above mentioned medicine(s) instead of going to a Doctor for your complaint(s)? (You may choose more than one option)**

- Saves Time
- Saves money
- To avoid the hassle of going to the doctor
- The same medicine successfully resolved my complains previously
- The same medicine worked for my friends/family members with similar complaints
- Left over medicine was present at home
- Other (please specify)

13. **Did you choose the medicine(s) yourself?**

- Yes
- No

**If NO, who suggested the medicine(s) to you?**

- A friend
- Parents
- Pharmacist
- Others (please specify)

**The following questions are regarding the adverse effects of antibiotics:**

14. **Do you know what Antibiotics are?**

- Yes
- No

15. **Are you aware that antibiotics can cause adverse effects?**

- Yes
- No

16. **Have you ever experienced any adverse effects after taking antibiotics?**

- Yes
- No
- I don’t know

**If YES, what symptoms did you experience?** ____________________________________________________________________________________________________________________________________________________________________

17. **Which of the following adverse effects do you know can be caused by antibiotics? (Tick as many as applicable)**

- Diarrhea / Abdominal pain
- Nausea / vomitting
- Allergic reactions
- Yellow eyes or skin
- Tiredness or dizziness
- Heart beat abnormalities
- Headache
- Fits / convulsions
- Eye problems
- Decreased hearing
- Fever
- Kidney problems
- Liver problems
- Unusual bleeding/bruising
- Teeth discoloration
- Muscle and joint pain
- Numbness or tingling in limbs
- Sleep problems

18. **Have you heard of the term “Antibiotic Resistance?”**

- Yes
- No
- Maybe

**19. What do you think happens to “Antibiotic Resistance” with indiscriminate un-prescribed use of antibiotics?**

- Increases
- Decreases
- Stays the same
- I don’t know.
